# Insulin-induced gene 2 alleviates ischemia-reperfusion injury in steatotic liver by inhibiting GPX4-dependent ferroptosis

**DOI:** 10.1038/s41420-025-02406-y

**Published:** 2025-04-01

**Authors:** Yichao Wu, Changbiao Li, Di Lu, Kangchen Chen, Renyi Su, Shengjun Xu, Fengqiang Gao, Zhengxing Lian, Fan Yang, Jun Chen, Fangqiang Wei, Xiao Xu, Zhikun Liu

**Affiliations:** 1 Department of Hepatobiliary, Pancreatic and Minimal Invasive Surgery, Zhejiang Provincial People’s Hospital (Affiliated People’s Hospital), Hangzhou Medical College, Hangzhou, China; 2 NHC Key Laboratory of Combined Multi-Organ Transplantation, Hangzhou, China; 3 Department of Gastrointestinal-Pancreatic Surgery, Zhejiang Provincial People’s Hospital (Affiliated People’s Hospital), Hangzhou Medical College, Hangzhou, China; 4 Institution of Clinical Medicine, Hangzhou Medical College, Hangzhou, China; 5 Department of Hepatobiliary and Pancreatic Surgery, Hangzhou First People’s Hospital, Hangzhou, China; 6 Zhejiang University School of Medicine, Hangzhou, China

**Keywords:** Transcriptomics, Metabolic disorders

## Abstract

Hepatic steatosis significantly elevates the vulnerability of the graft to ischemia-reperfusion (I/R) injury during liver transplantation (LT). We investigated the protective role of insulin-induced gene 2 (Insig2) in steatotic liver’s I/R injury and underlying mechanisms. Employing mouse model with Insig2 knock-out or hepatocyte-specific overexpression and high-fat diets to induce steatosis, we subjected these mice to hepatic I/R injury. The primary hepatocytes isolated from steatotic liver were used in in vitro hypoxia/reoxygenation (H/R) experiment. Our integrated in vivo *and* in vitro approach uncovered that Insig2 deficiency exacerbated steatotic liver’s damage following hepatic I/R injury, whereas its overexpression offers protection. Mechanically, integrative analysis of transcriptome, proteome, and metabolome found that Insig2 deficiency disturbed lipid metabolism and oxidative stress homeostasis, particularly inhibiting GPX4 expression to induce ferroptosis. Furthermore, chemical inhibition of ferroptosis reversed the deleterious effect of Insig2 deficiency; whereas the protective influence of Insig2 overexpression was negated by the target inhibition of GPX4, leading to an exacerbation of hepatic I/R damage. These insights underscored the potential of the Insig2-GPX4 axis as a therapeutic target, presenting a novel avenue for enhancing the resilience of steatotic liver grafts against I/R injury.

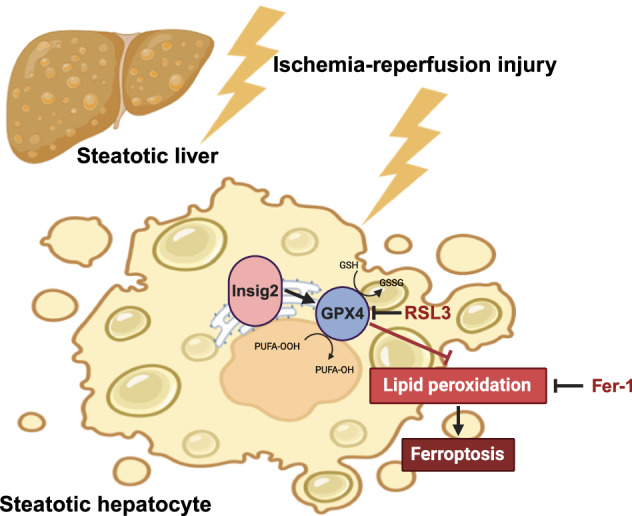

## Introduction

Liver transplantation (LT) continues to stand out as the most efficacious treatment for individuals afflicted with acute liver failure, end-stage liver disease, or advanced liver cancer [1, 2]. Given the increasing gap between organ availability and demand, there is an inevitable trend in utilizing marginal liver donors, which encompass older donors, donors after cardiac death, and steatotic donors [3]. Approaches to enhance the viability of these grafts may significantly expand the pool of donors eligible for LT. With the growing morbidity of non-alcoholic fatty liver disease (NAFLD) worldwide, steatotic liver has become increasingly common in donor organs [4, 5]. The presence of steatosis renders the liver more sensitive and less tolerant to ischemia-reperfusion (I/R) injury, as lipid deposition in hepatocytes complicates the underlying pathophysiologic mechanism [6, 7]. The utilization of steatotic graft was at a higher risk for allograft dysfunction and primary nonfunction [8]. Current studies have shown that liver steatosis greater than 10% is associated with early allograft dysfunction, long-term graft loss, and tumor recurrence [9]. Several interventions aimed at mitigating preservation injury in steatotic livers have been explored in prior studies [10]. Nonetheless, novel graft preservation solutions and machine perfusion techniques have yet to realize their benefits in clinical practice. In-depth investigations into the mechanisms underlying I/R injury in the context of steatotic liver are crucial for identifying therapeutic strategies to ensure graft safety and further expand the donor pool.

Ferroptosis was reported to contribute to liver injury and dysfunction in LT [11]. Ferroptosis is characterized by excessive lipid peroxide buildup and disrupted cellular redox equilibrium, which often involves the impaired function of the lipid repair enzyme glutathione peroxidase 4 (GPX4), significant iron accumulation and the peroxidation of polyunsaturated fatty acids (PUFAs) [12, 13]. Furthermore, ferroptosis in hepatocytes has been recognized the major cell death type involved in steatotic liver I/R injury [14, 15].

Insulin-induced gene 2 (Insig2) is an integral protein located in the endoplasmic reticulum and is modulated by insulin signals. Its primary function involves regulating the activity of sterol regulatory element-binding protein 1c (SREBP-1c), thereby participating in the synthesis of cholesterol and fatty acids [16, 17]. Additionally, Insig2 plays a crucial role as a regulator of energy production and cellular immune metabolism [18–20]. Insig2 was recently characterized to be involved in detection of abnormal lipids and reactive oxygen species (ROS), and in maintenance of intracellular homeostasis [21, 22]. Furthermore, our previous research revealed Insig2 protected hepatic I/R injury via remolding glucose metabolism [23]. Insig2 modulated the translocation of SCAP-SREBP1 and the activation of SREBP1, which ultimately led to the increased transcription of stearoyl-CoA desaturase 1, thereby inhibiting ferroptosis [24]. However, the function of Insig2 in steatosis graft injury remains to be elucidated.

Considering the role of Insig2 in preserving intracellular immune and metabolic balance, we hypothesized that Insig2 could likewise exert a protective effect in steatosis hepatic I/R injury, and also aimed to investigate the mechanisms underlying this effect as well as the therapeutic possibilities for marginal donor recovery.

## Results

### Hepatic Insig2 expression is downregulated during I/R or H/R in steatotic liver

**Fig. 1:**
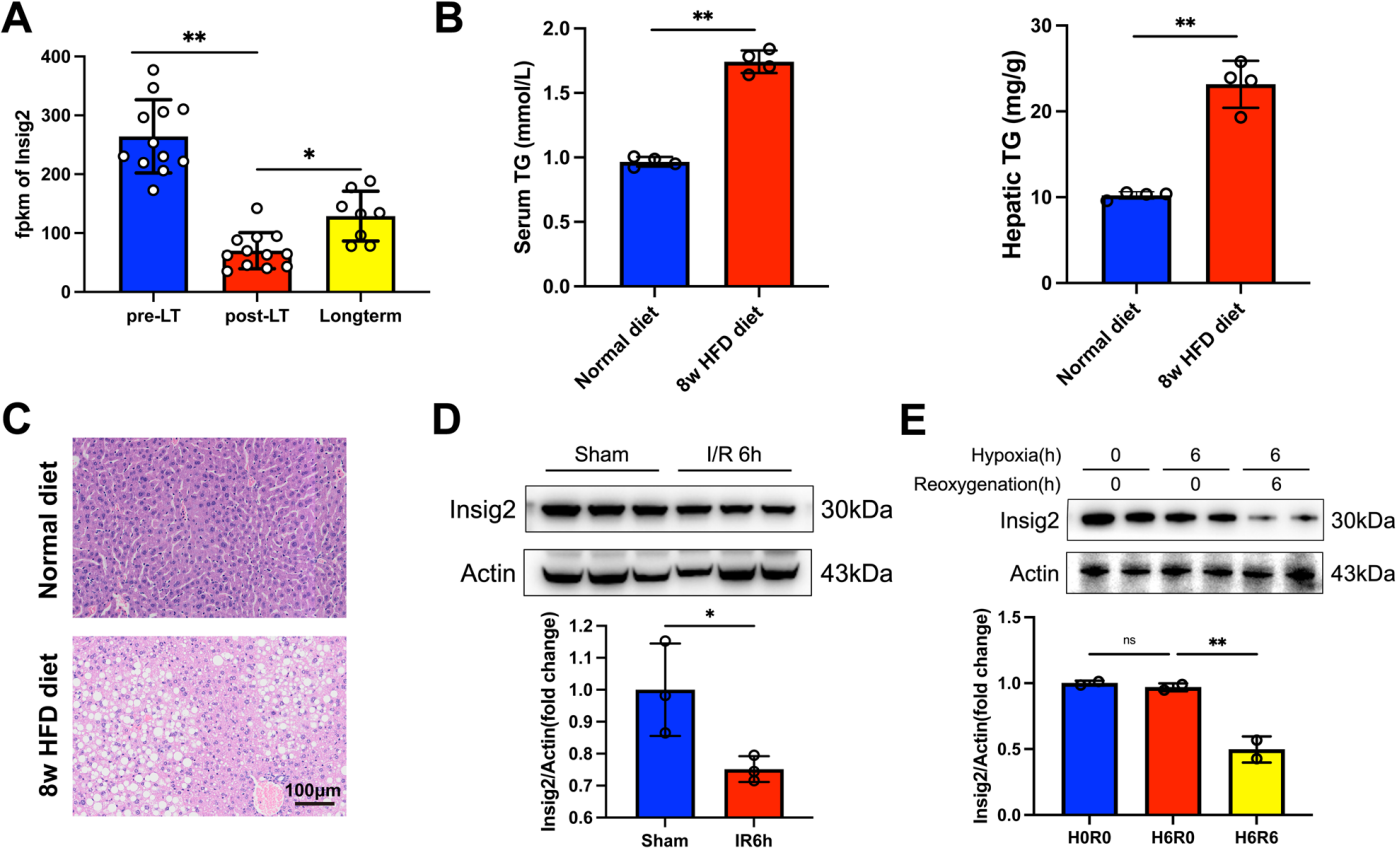
Expression of Insig2 is down-regulated after I/R injury in steatotic liver. **A** The change of Insig2 mRNA levels in mice orthotopic liver transplantation with steatotic graft from GSE193764 Database. **B**, **C** The serum and liver tissue TG levels in mice fed HFD for 8 weeks or normal diets (*n* = 4/group). **D** Western blotting analysis of endogenous Insig2 expression in the steatotic liver subjected to sham operation or I/R injury (*n* = 3/group). **E** Western blotting analysis of Insig2 protein expression in primary hepatocytes with steatosis subjected to H/R stimulation (*n* = 2/group). *p < 0.05, **p < 0.01.

In the rat model of steatosis LT, we observed the transcriptional expression levels of Insig2 in the transplanted liver were significantly decreased during LT (GSE193764, Fig. 1A). We aimed to validate this finding in mouse model of hepatic I/R injury. After 8 weeks of HFD diet feeding, mice exhibited elevated serum and hepatic TG levels compared to chow diet (Fig. 1B). The H&E staining revealed moderate hepatic steatosis, characterized by the presence of lipid vacuoles [10, 25] (Fig. 1C). The HFD mice were subjected to 1.5 h of ischemia, followed by 6 h of reperfusion. Moreover, the steatosis primary hepatocytes were isolated from the HFD mice’s liver, and subjected to 6 h of hypoxia, followed by 6 h of oxygenation. The endogenous expression of Insig2 protein was evaluated by western blot. As anticipated, the results demonstrated that I/R injury downregulated Insig2 expression in steatosis hepatocytes (Fig. 1D, E).

### Insig2 knockout in mice with diet-induced steatotic liver aggravates liver damage following I/R injury

**Fig. 2:**
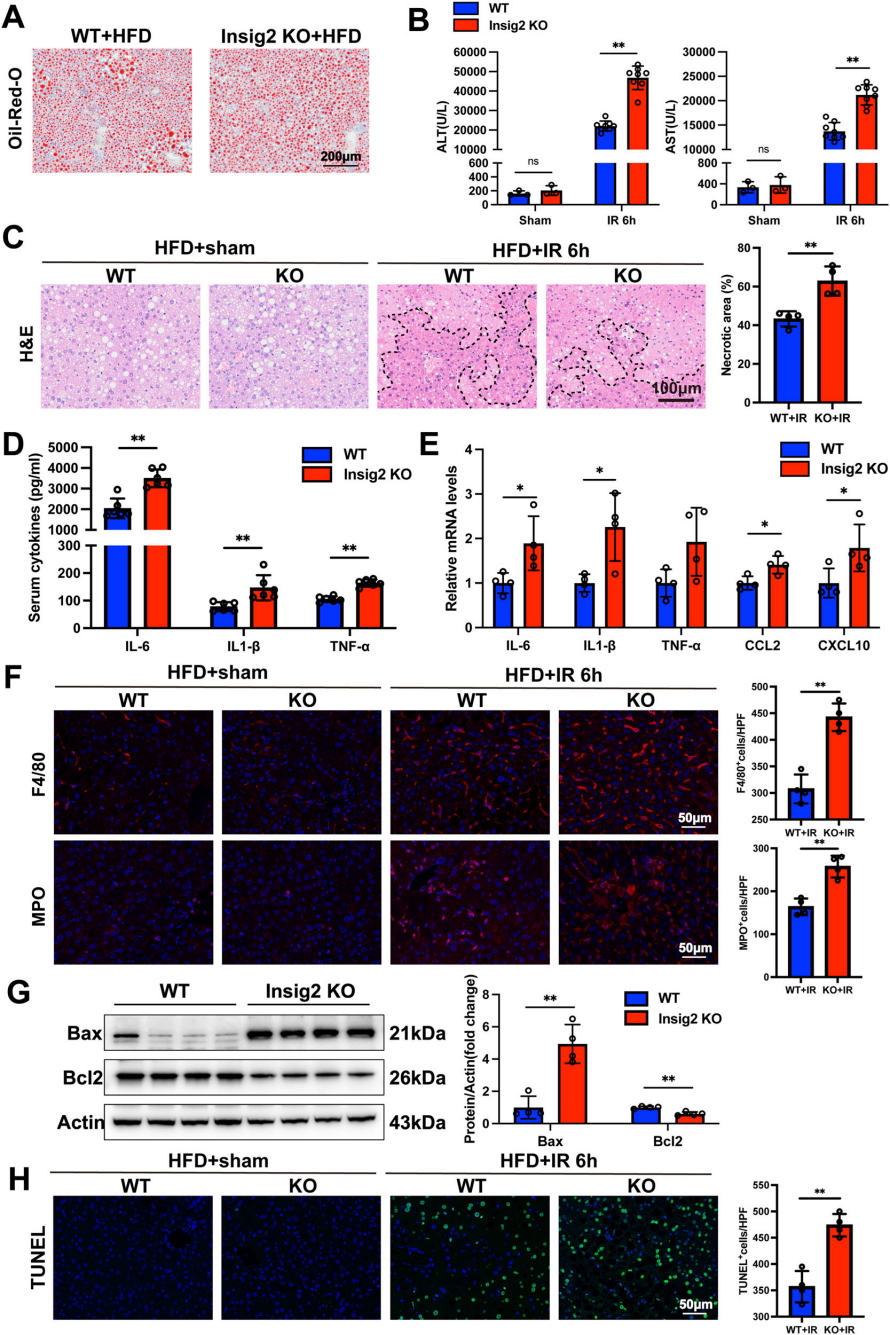
Insig2 deficiency exacerbates hepatic I/R injury in steatotic liver. **A** Oil Red O staining of liver sections in WT and Insig2-KO mice fed with HFD for 8 weeks. **B**. The serum ALT and AST levels in WT and Insig2-KO mice with steatotic liver following sham (*n* = 3/group) and I/R surgery (*n* = 8/group). **C** Representative H&E staining liver sections from liver tissues of WT and Insig2-KO mice with steatitic liver following sham operation and I/R injury (*n* = 4/group). **D** The serum levels of inflammatory cytokines (*IL-6, IL-1β, TNF-α*) in WT and Insig2-KO mice with steatotic livers following I/R (*n* = 6/group). **E** qPCR analysis of the mRNA levels of proinflammatory cytokines and chemokines (*IL-6, IL-1β, TNF-α, Ccl2, and Cxcl10*) in WT and Insig2-KO mice with steatotic livers following I/R (*n* = 4/group). **F** Representative F4/80 and MPO IF staining (red) and statistics in liver sections of WT and Insig2-KO mice following sham or I/R surgery (*n* = 4/group). **G** Western blotting analysis of endogenous apoptosis-related protein expression in the steatotic liver subjected to I/R injury (*n* = 4/group). **H** TUNEL staining (green) and statistics in liver sections of WT and Insig2 KO mice following sham or I/R surgery (*n* = 4/ group). *p < 0.05, **p < 0.01.

Firstly, Insig2 KO and WT mice were fed with HFD diet for 8 weeks, all mice developed moderate hepatic steatosis. We found that the hepatic TG accumulation evidenced by Oil Red O staining was not different by Insig2 deficiency (Fig. 2A). To document the role of Insig2 on steatosis hepatic I/R injury, Insig2 KO and WT mice with steatotic liver were subjected to I/R surgery. The serum ALT and AST levels were markedly elevated in Insig2 KO mice compared to WT controls (Fig. 2B). Liver necrosis area, indicated by H&E staining, was also significantly greater in Insig2 KO mice (Fig. 2C). Besides, Insig2 deficiency was associated with increased levels of inflammatory cytokines (*IL-6, IL-1β, TNF-α*) in serum and heightened hepatic mRNA expression of proinflammatory cytokines and chemokines (*IL-6, IL-1β, TNF-α, Ccl2, Cxcl10*), as depicted in Fig. 2D, E. IF assays revealed heightened infiltration degree of F4/80 and MPO-positive cells in Insig2 KO liver (Fig. 2F). Furthermore, we quantified the expression of apoptosis-related proteins in Insig2 KO and WT liver subjected to I/R injury. Our results demonstrated that Insig2 KO increased pro-apoptotic protein *Bax* and decreased anti-apoptotic *Bcl2* in KO mice (Fig. 2G). TUNEL staining supported these findings that Insig2 KO exacerbated cell apoptosis in steatotic liver post I/R injury (Fig. 2H).

### Hepatocyte-specific Insig2 overexpression alleviated liver steatosis damage following I/R injury

**Fig. 3:**
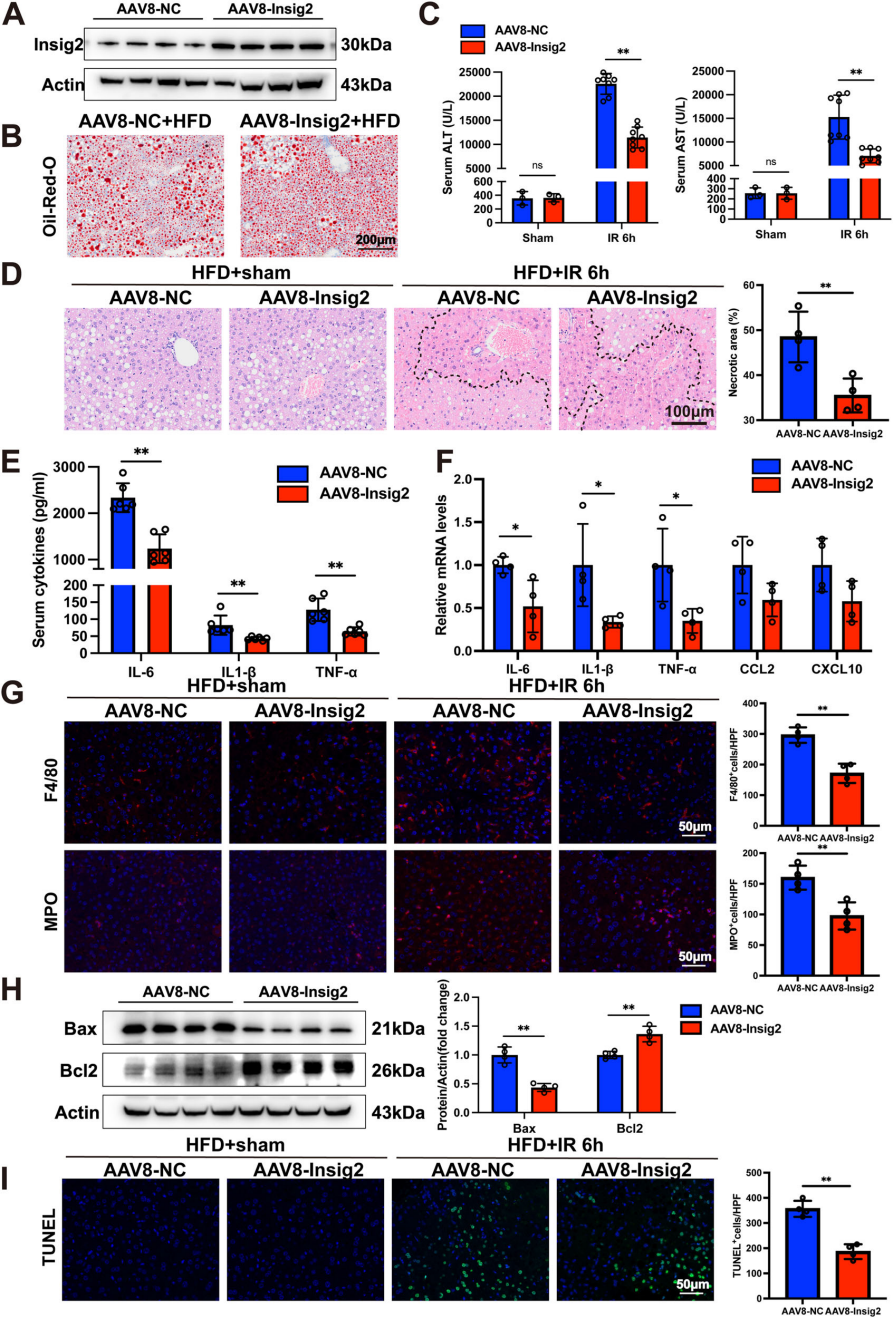
Hepatic Insig2 overexpression ameliorates hepatic I/R injury in steatotic liver. **A** Western blot analysis of AAV8-Insig2 and AAV8-NC transfection efficiency in steatotic liver. **B** Oil Red O staining of liver sections in AAV8-NC and AAV8-Insig2 mice fed with HFD for 8 weeks. **C** The serum ALT and AST levels in AAV8-NC and AAV8-Insig2 mice with steatotic liver following sham (*n* = 3/group) and I/R surgery (*n* = 8/group). **D** Representative H&E staining liver sections from liver tissues of AAV8-NC and AAV8-Insig2 mice with steatitic liver following sham operation and I/R injury (*n* = 4/group). **E** The serum levels of inflammatory cytokines (*IL-6, IL-1β, TNF-α*) in AAV8-NC and AAV8-Insig2 mice with steatotic livers following I/R (*n* = 6/group). **F** qPCR analysis of the mRNA levels of proinflammatory cytokines and chemokines (*IL-6, IL-1β, TNF-α, Ccl2, and Cxcl10*) in AAV8-NC and AAV8-Insig2 mice with steatotic livers following I/R (*n* = 4/group). **G** Representative F4/80 and MPO IF staining (red) and statistics in liver sections of AAV8-NC and AAV8-Insig2 mice following sham or I/R surgery (*n* = 4/group). **H** Western blotting analysis of endogenous apoptosis-related protein expression in the steatotic liver subjected to I/R injury (*n* = 4/group). **I** TUNEL staining (green) and statistics in liver sections of AAV8-NC and AAV8-Insig2 mice following sham or I/R surgery (*n* = 4/ group). *p < 0.05, **p < 0.01.

To further delineate the functional role of Insig2 in hepatic I/R injury with steatosis, hepatocyte-specific Insig2 overexpression (Insig2 OE) mice were generated via AAV8-Insig2 intravenous injection, with AAV8-control-infected mice as parallel controls. After 8 weeks of HFD diet feeding, Insig2 OE and control mice with steatotic liver were established and successfully confirmed (Fig. 3A). Prior to I/R, hepatic TG accumulation remained unchanged (Fig. 3B). At 6 h post-I/R, Insig2 OE mice exhibited significantly lower serum ALT and AST levels compared to controls (Fig. 3C), along with reduced hepatic necrosis (Fig. 3D). Furthermore, Insig2 OE mice displayed decreased levels of the secretion and mRNA expression of proinflammatory factors, reduced inflammatory cell infiltration in liver (Fig. 3E–G). Western blot and TUNEL staining revealed that Insig2 OE significantly mitigated apoptosis and modulated apoptotic protein expression (Fig. 3H, I). Collectively, these findings indicated that Insig2 overexpression ameliorated hepatic damage, inflammation, and apoptosis during I/R in steatotic livers.

### Insig2 mitigated inflammation and apoptosis in steatotic hepatocytes from H/R stimulation

**Fig. 4:**
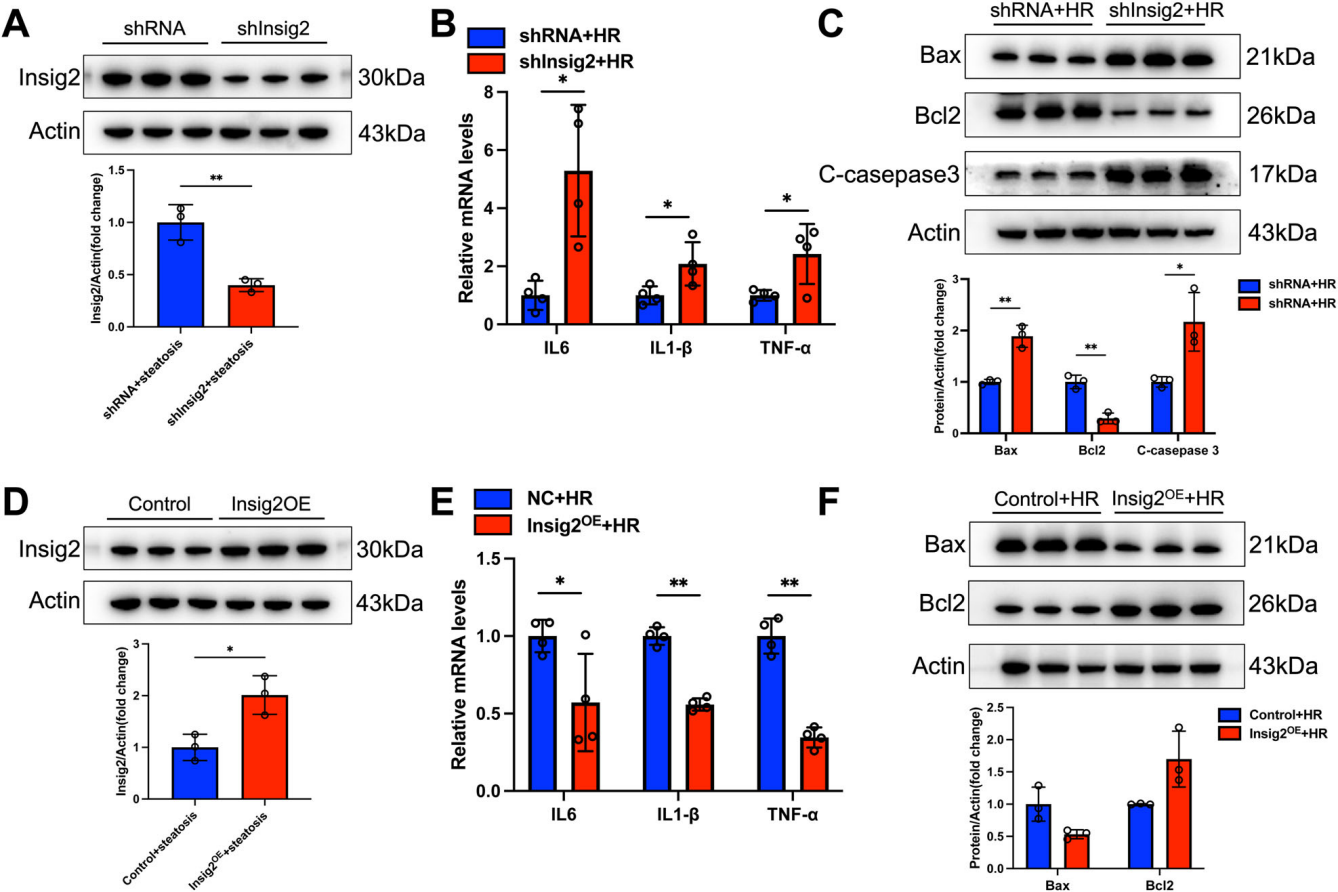
Insig2 alleviates primary hepatocytes inflammation and apoptosis during H/R. **A** Insig2 protein expression in primary hepatocytes extracted from mice infected with shRNA or shInsig2 AAV8 followed by incubation of OA:PA. **B** mRNA levels of proinflammatory factors (*IL-6, IL-1β, TNF-α*) in Insig2-knockdown hepatocytes with steatosis after H/R stimulation (*n* = 3/group). **C** Expression of apoptosis-related proteins in Insig2-knockdown hepatocytes with steatosis after H/R stimulation (*n* = 3/group). **D** Insig2 protein expression in primary hepatocytes extracted from mice infected with control or Insig2 overexpressing AAV8 followed by incubation of OA:PA. **E** mRNA levels of proinflammatory factors in Insig2-overexpression hepatocytes with steatosis after H/R stimulation (*n* = 3/group). **F** Expression of apoptosis-related proteins in Insig2-overexpression hepatocytes with steatosis after H/R stimulation (*n* = 3/group). *p < 0.05, **p < 0.01.

Primary hepatocytes, derived from mice treated with AAV8 for either Insig2 knockdown or overexpression at a uniform age, were treated with OA:PA and then exposed to H/R stimulation (Fig. 4A–D). Consistent with in vivo findings, Insig2 knockdown hepatocytes under steatotic conditions exhibited markedly increased levels of inflammatory cytokines (*IL-6, IL-1β, TNF-α*) post-H/R (Fig. 4B). Additionally, pro-apoptotic proteins (*Bax, Cleaved-caspase 3*) were upregulated, whereas the anti-apoptotic protein (*Bcl2*) was downregulated in these knockdown cells (Fig. 4C). Conversely, hepatocytes with Insig2 overexpression showed significantly diminished inflammation and apoptosis levels (Fig. 4E, F).

### Multi-omics analysis indicates that Insig2 regulated lipid remodeling and ferroptosis in steatosis I/R injury

**Fig. 5:**
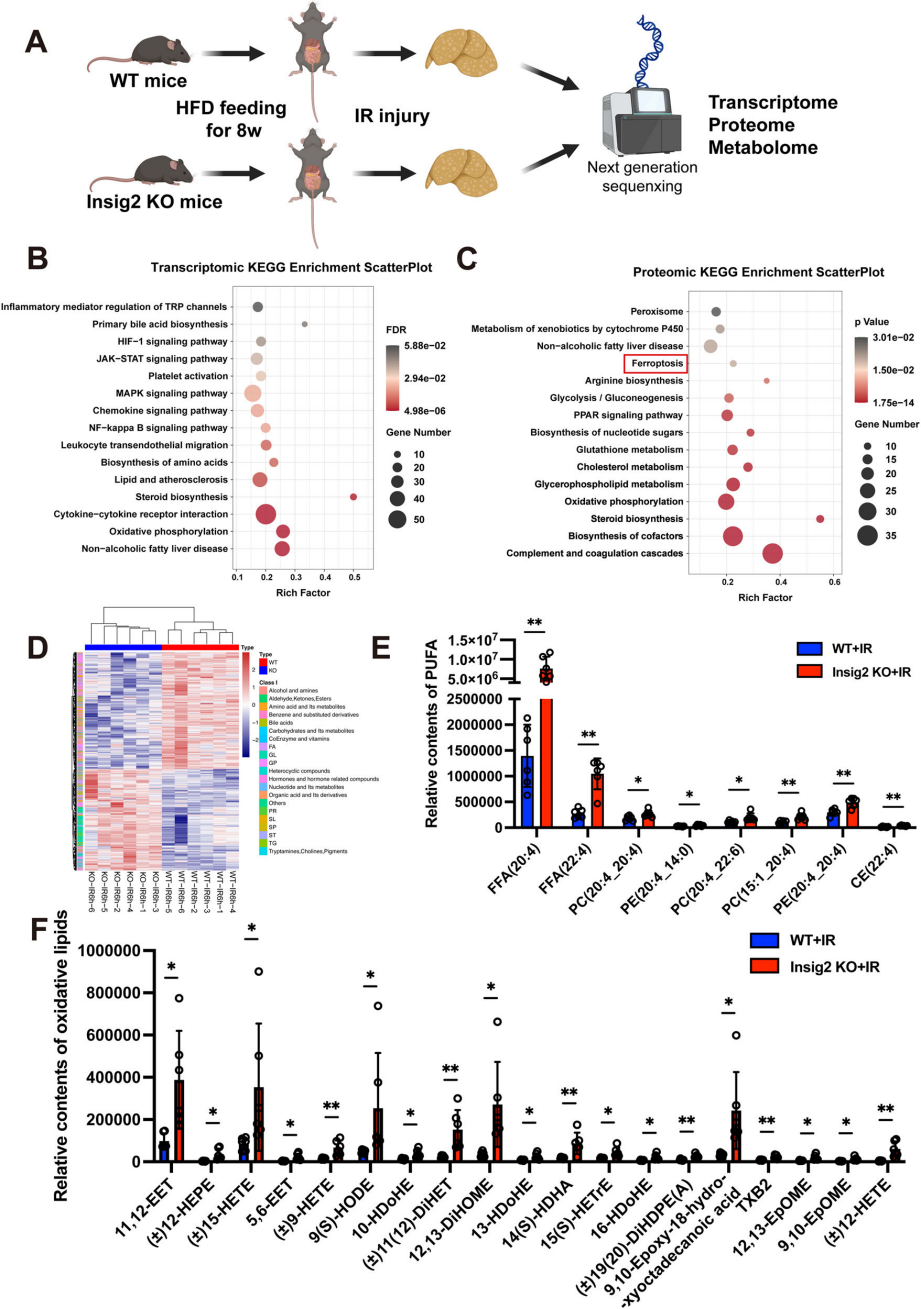
Transcriptome, proteome, and metabolome integrative analysis reveals disturbed pathways in Insig2-KO mice with steatotic liver subjected to I/R injury. **A** The experimental design of the muti-omics approach. **B** The bubble plot of KEGG pathway enrichment of differentially expressed genes. **C** The bubble plot of KEGG enrichment pathway of differential expressed proteins. **D** The heatmaps of dysregulated metabolites in metabolomic analysis. **E** The substrate levels of oxidized phospholipids that contained polyunsaturated fatty acids (PUFAs) chains (arachidonic acid (ArA) and adrenic acid (AdA)) in metabolomic analysis (*n* = 6/group). **F** The levels of oxidized lipids in metabolomic analysis (*n* = 6/group). *p < 0.05, **p < 0.01.

To explore the mechanisms underlying Insig2’s role in steatotic hepatic I/R injury, a multi-omics strategy was employed, analyzing transcriptomic, proteomic, and metabolomic data from steatotic liver tissues of Insig2 KO and WT mice post-I/R (Fig. 5A). The hierarchical clustering and volcano mapping delineated the profiles of DEGs and DEPs (Fig. S1). The KEGG enrichment analysis revealed significant enrichment of lipid metabolism and ferroptosis pathways in both transcriptomic and proteomic data (Fig. 5B, C). Additionally, the GO pathway analysis identified various differential pathways related to intermediary metabolism, oxidoreductase activity, and immune response (Fig. S2).

The metabolome was particularly insightful in characteristic of ferroptosis, as it detected altered metabolites that were directly involved in iron metabolism and lipid peroxidation process. The metabolomic analysis uncovered pronounced disparities in lipid profiles between Insig2 KO and WT mice, with a notable increase in fatty acids (FAs), phosphatidylethanolamines (PEs) and phosphatidylcholines (PCs) in Insig2 KO mice with steatotic livers (Fig. 5D). Further evaluation of substrates of PUFAs peroxidation and products of oxidative lipids, revealed elevated levels of oxidized arachidonic acid (ArA) and adrenic acid (AdA) metabolites, notably 12-HETE and 15-HETE (Fig. 5E, F). Collectively, these findings indicated that Insig2 deficiency disrupted lipid metabolism and enhanced ferroptosis, exacerbating hepatic I/R injury in the context of steatosis.

### Insig2 protects against steatosis I/R injury partly by inhibiting ferroptosis

**Fig. 6:**
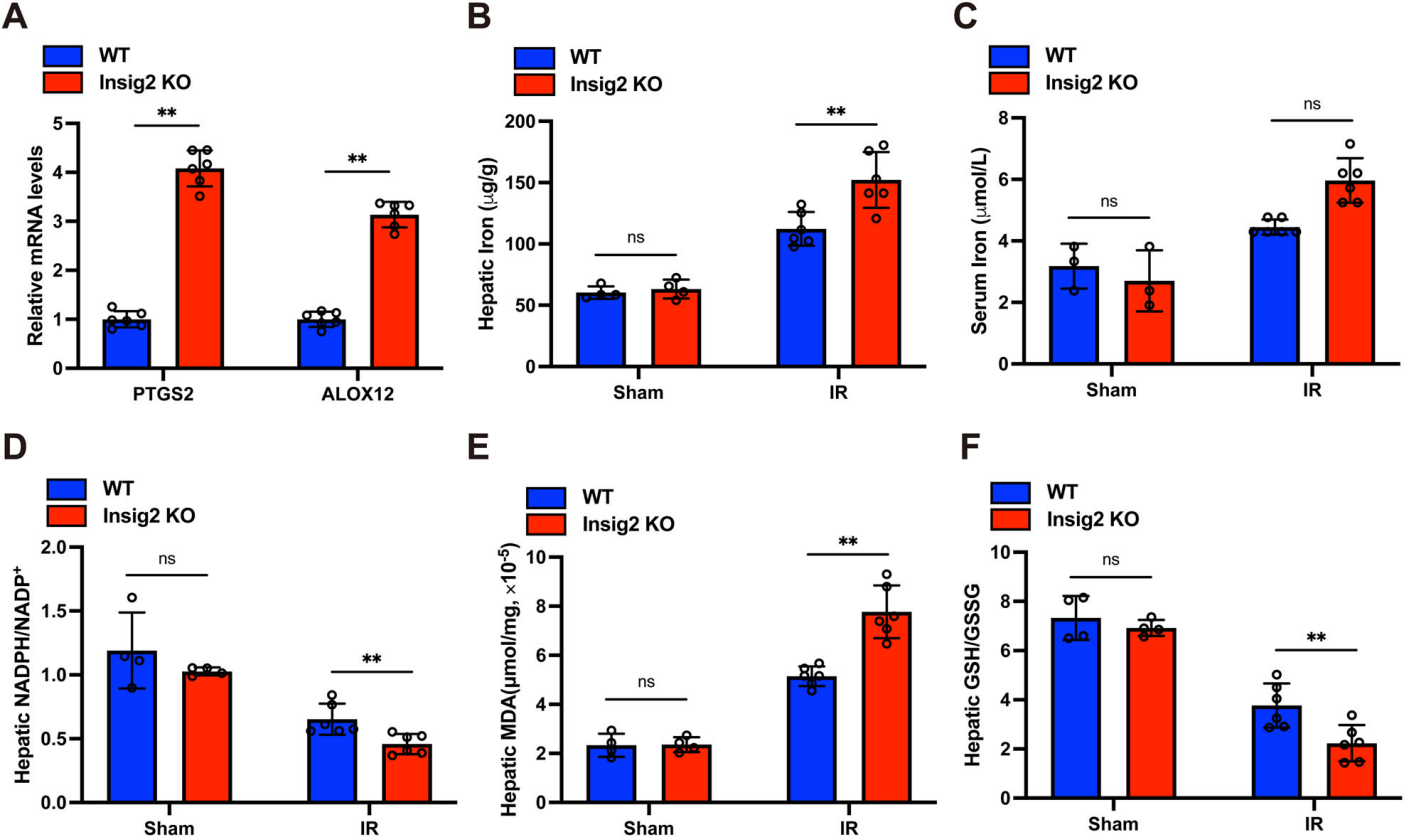
Insig2 defiency facilitates I/R-induced ferroptosis in steatotic liver. **A** Relative PTGS2 and ALOX12 mRNA levels in liver (*n* = 6/group), (**B**, **C**). Iron contents in liver tissue and serum (*n* = 6/group), (**D**–**F**). Hepatic levels of NADPH/NADP^+^, MDA, and GSH/GSSG (*n* = 6/group) were detected at sham and I/R groups from Insig2 KO and WT mice with steatotic liver. *p < 0.05, **p < 0.01.

To ascertain Insig2’s role in ferroptosis, we measured serum and hepatic iron levels, the NADPH/NADP^+^ and GSH/GSSG ratios, MDA levels, and mRNA expressions of ferroptosis biomarkers (*PTGS2, ALOX12*) in the liver tissues. We found that the elevated iron concentrations in serum and liver tissues (Fig. 6A, B), increased *PTGS2* and *ALOX12* expression (Fig. 6C), and higher hepatic MDA levels (Fig. 6D) were observed in Insig2 KO mice following steatosis I/R injury compared to WT mice. By contrast, the hepatic NADPH/NADP^+^ and GSH/GSSG ratios were diminished, indicating an attenuated antioxidant capacity in Insig2 KO mice (Fig. 6E, F).

**Fig. 7:**
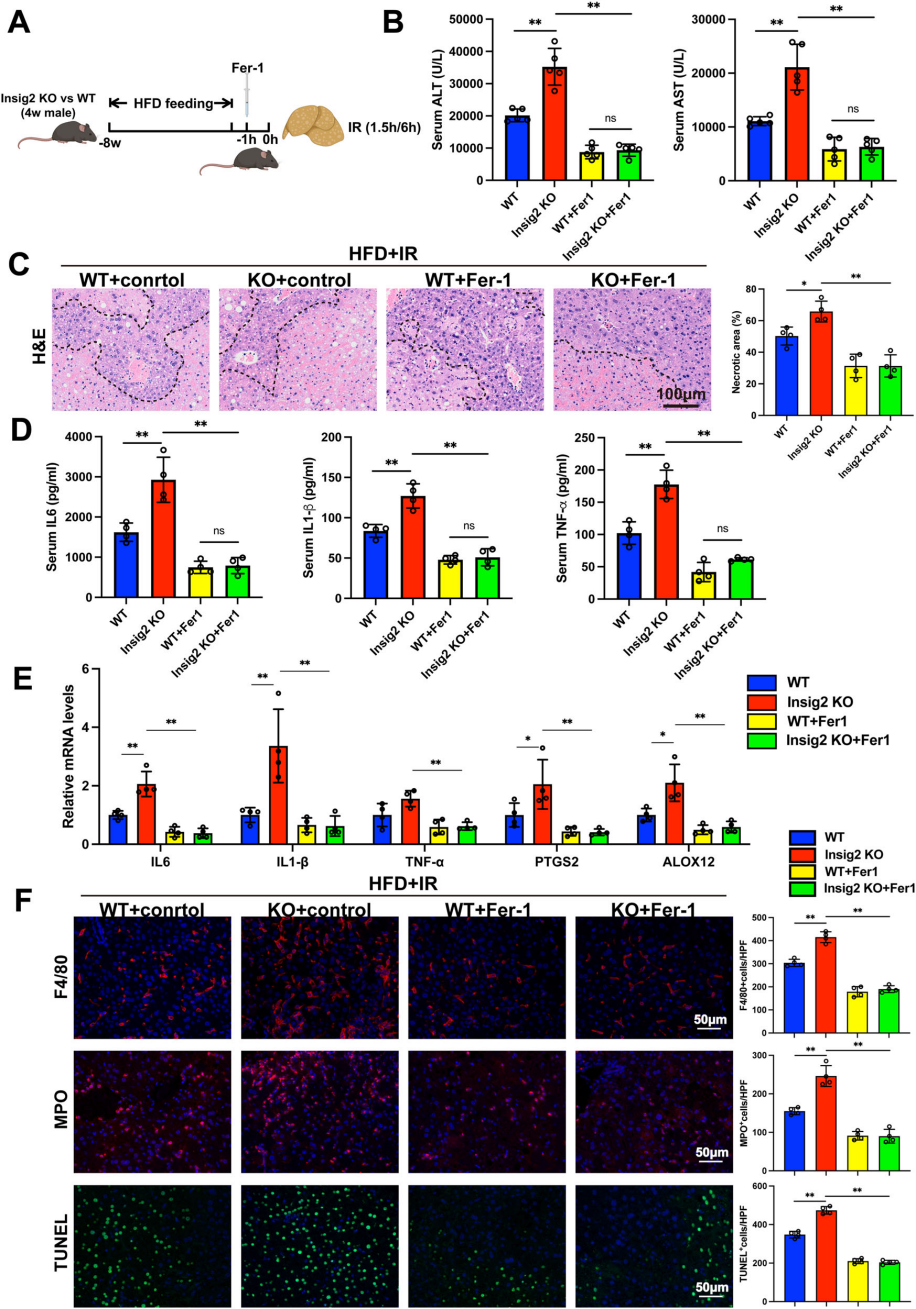
Insig2 protects against I/R injury in steatotic liver by partly regulating ferroptosis. **A** Graphical representation of experimental design. Fer-1 was i.v. injected 1 h before I/R of Insig2 KO and control WT mice with steatotic liver to inhibit ferroptosis. **B** The serum ALT and AST levels in the indicated group (n = 5/group). **C** Representative H&E staining liver sections from indicated group (*n* = 4/group). **D** The serum levels of inflammatory cytokines (*IL-6, IL-1β, TNF-α*, *n* = 4/group). **E** qPCR analysis of the mRNA levels of proinflammatory cytokines (*IL-6, IL-1β, TNF-α*, *n* = 4/group) and ferroptosis indicator (*PTGS2, ALOX12*, *n* = 4/group). **F** Representative F4/80 and MPO IF staining and TUNEL staining in liver sections (*n* = 4/group). *p < 0.05, **p < 0.01.

To further establish if Insig2 protected steatotic liver against I/R injury partly through the inhibition of ferroptosis, we administrated Fer-1 (a selective ferroptosis inhibitor) to Insig2 KO and WT mice before I/R surgery. As anticipated, Fer-1 preconditioning successfully rescued the exacerbating effects of Insig2 deficiency on I/R-induced liver damage (Fig. 7). Moreover, the pro-inflammatory and pro-apoptotic effects enhanced by the deficiency of Insig2 were significantly restored by Fer-1 (Fig. 7).

### Insig2 inhibits GPX4-dependent ferroptosis in steatosis I/R injury

**Fig. 8:**
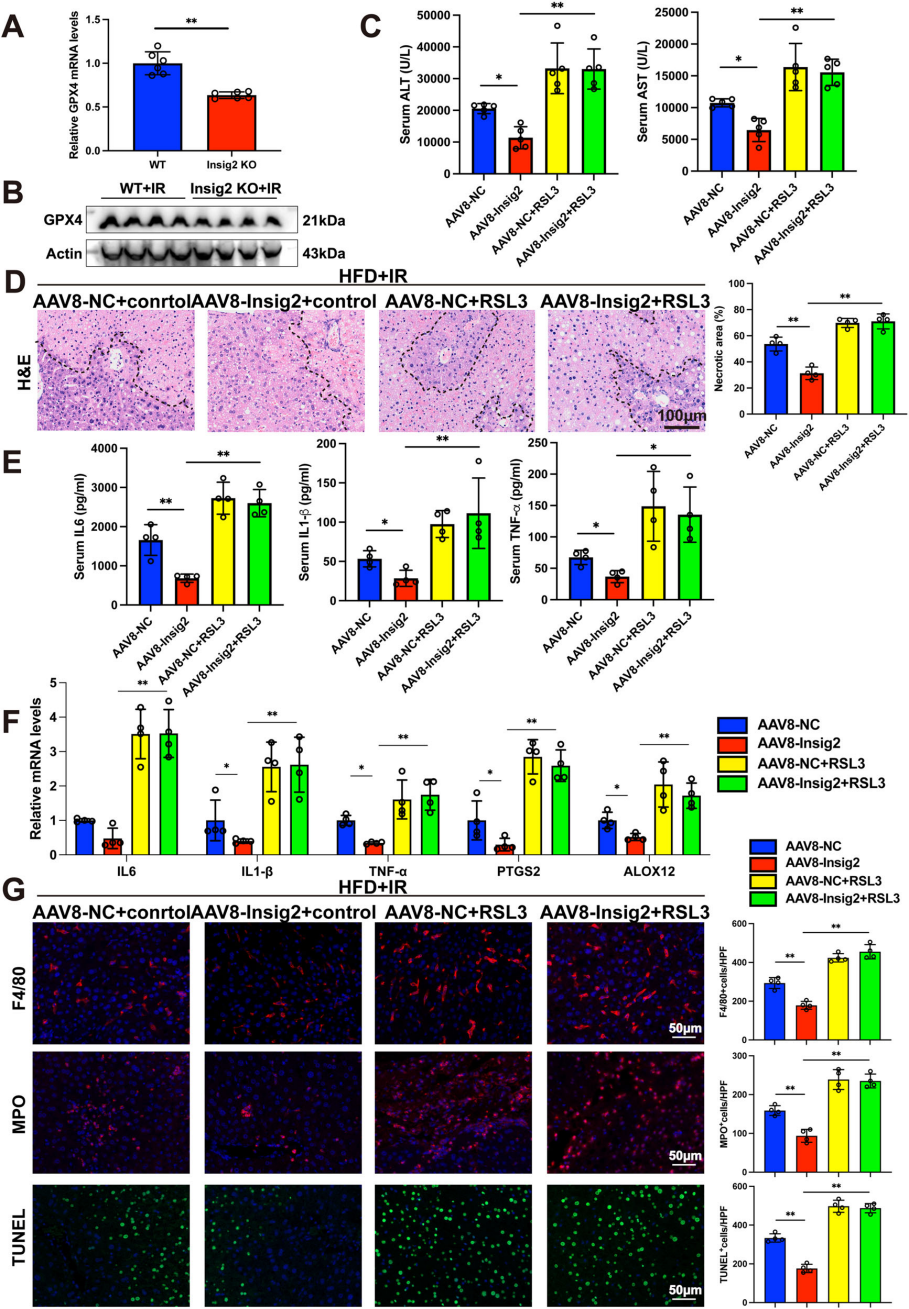
Insig2 regulates I/R injury by inhibiting GPX4-dependent ferroptosis in steatotic liver. **A**, **B** The mRNA and protein levels of GPX4 were evaluated in livers from WT and Insig2 KO mice with steatotic liver (*n* = 6/group for mRNA samples, *n* = 4/group for protein samples). **C**–**G** RSL3 was i.p. injected 1 h before I/R of Insig2 OE and control mice with steatotic liver to inhibit GPX4. The serum ALT and AST (**C**, n = 5/group), necrosis areas in liver section (**D**, *n* = 4/group), serum inflammatory cytokines (**E**, *n* = 4/group), expression of proinflammatory cytokines and ferroptosis indicator (**F**, *n* = 4/group), Representative F4/80 and MPO IF staining and TUNEL staining in liver sections (**G**, *n* = 4/group). *p < 0.05, **p < 0.01.

Further, we explored how Insig2 affected ferroptosis. Among all key genes and proteins in the ferroptosis pathways, we found that GPX4 was both down-regulated in Insig2 KO mice compared to WT mice (Fig. S3). We confirmed a significant reduction in hepatic GPX4 expression in Insig2-deficient livers, suggesting a mechanism by which Insig2 modulates GPX4 level in steatotic liver (Fig. 8A, B). Furthermore, the AAV8-Insig2 viruses were injected into 8-week HFD-fed mice, then RSL3 (a competitively GPX4 binding inhibitor) was administrated to confirm the role of GPX4 in Insig2 OE-induced steatotic liver I/R damage (Fig. S4). After hepatic I/R injury, RSL3 significantly reversed the protective effect of Insig2 overexpressing (Fig. 8C, D). To illustrate, RSL3 administration activated the ferroptosis, upregulated the expression of inflammatory cytokines, and accelerated cell apoptosis (Fig. 8E–G). In summary, these results indicated that Insig2 could suppress ferroptosis in a GPX4-dependent manner to protect against steatotic liver injury under I/R.

## Discussion

In the face of the high mortality on the liver transplant waitlist and the scarcity of liver donors, donors with steatosis are frequently considered for use as marginal grafts. Growing evidence supported that mild-moderate macrosteatotic grafts could be successfully used for LT with appropriate donor-recipient matching [26]. However, numerous studies have indicated that the presence of hepatic steatosis was prone to I/R injury, thereby raising the potential for poor post-transplant complications [27, 28]. Factors such as mitochondrial dysfunction, metabolism disorder, lowered antioxidant capacity, and amplified inflammatory response were believed to contribute to the increased vulnerability of steatotic livers to I/R injury [9, 29]. There is an urgent demand for treatments that can mitigate the harmful effects of I/R injury in steatotic livers. In our study, for the first time, we examined Insig2’s protective effects and underlying mechanisms against steatotic liver I/R injury, providing valuable insights for devising strategies to mitigate graft damage when utilizing steatotic donors.

Insig2 is essential in the body’s reaction to insulin and a variety of biological signals, exhibiting widespread expression throughout numerous tissues, with a particularly heightened presence in hepatocytes of the liver [30, 31]. Extensive research has highlighted Insig2’s significant involvement in maintaining glucose and lipid balance, primarily through the Insig-SREBP pathway [32–34]. Furthermore, several studies have underscored Insig-SREBP’s regulatory importance in cell death, redox equilibrium, and inflammatory response, which were closely related to I/R injury [21, 22, 35]. In our study, the expression of Insig2 was found to be significantly downregulated in both rat liver transplantation and mouse model of I/R injury in steatotic liver, suggesting its potential role in regulating steatosis hepatic I/R injury. Subsequently, we utilized Insig2 knockout and hepatocyte-specific Insig2 overexpression mice to investigate its biological role in steatotic I/R injury. By administering HFD to mice for 8 weeks, we successfully established a mouse model of liver steatosis with a macrosteatosis (MaS) degree ranging from ~30 to 60% [10, 25]. This model was utilized as a donor liver to simulate the clinical application of moderate MaS donor livers. Notably, we observed that neither Insig2 knockout nor overexpression altered the predominant determinant of hepatic steatosis during HFD feeding—TG. We speculated that the possible reason lies in the complementary roles of Insig1 and Insig2 in regulating lipids, particularly TG (Fig. S5) [36]. However, Insig2 did modulate the extent of liver damage, apoptosis, and inflammatory responses following I/R. This also indicated that lipid substances such as cholesterol and free fatty acids played a more significant role in the susceptibility to hepatic I/R injury [37]. To determine its downstream mechanism, transcriptomic and proteomics analysis suggested that Insig2 KO led to impairment of lipid and energy metabolic pathways, including ferroptosis. The present investigation provided evidence that Insig2 KO expedited iron accumulation, increased oxidative stress (MDA), and weakened the in vivo antioxidant defense systems (NADPH, GSH). Crucially, pharmacological inhibition of ferroptosis significantly counteracted the exacerbated outcomes associated with Insig2 deficiency in steatotic hepatic I/R injury. Hence, our research findings indicated that Insig2 protected steatotic liver damage induced by I/R through the partly suppression of ferroptosis in hepatocytes.

Ferroptosis, a form of cell death characterized by iron-mediated lipid peroxidation, assumes a pivotal role in the LT process and the advancement of hepatic I/R injury [38–40]. The condition of iron overload and lipid peroxidation stress are more commonly observed in steatosis hepatic I/R [15]. Jiao’s research indicated that ferroptosis is the predominant form of cell death occurring in hepatocytes in steatotic liver I/R injury [14]. Our previous study demonstrated that Insig2 could upregulate the pentose phosphate pathway (PPP), which is an important glucose metabolism pathway involved in the regulation of NADPH, GSH, and ROS [23]. Recent reports also revealed that PPP imposed potential regulatory roles in ferroptosis [41]. Given the interplay between lipid metabolism and iron homeostasis, Insig2 may indirectly influence the levels of PUFAs within the cell membrane. These PUFAs are the primary substrates for lipid peroxidation in the process of ferroptosis [42, 43]. Based on metabolome sequencing, the difference in the hepatic content of metabolites was been fully characterized. We found that the level of oxidative lipids, oxidized AA metabolites were significantly increased in Insig2 KO liver tissues upon I/R injury, which was in accordance with the result of transcriptome and proteome.

Additionally, we found that Insig2 and the ferroptosis inhibitor modulated the levels of cellular necrosis and apoptosis in liver. These mice exhibited similar trends in lipid peroxidation and ferroptotic activity, indicating a potential interplay among ferroptosis and different modes of cell death [12]. While these forms of cell death are distinct in their mechanisms, they can interact in complex ways. Studies have shown that lipid peroxides could regulate NF-κB and Casepase signals to initiate apoptosis [44]. To illustrate, under conditions of iron overload, cells may simultaneously undergo ferroptosis as well as apoptosis or necrosis. It is well-recognized that ROS generation and lipid peroxidation contribute to hepatic injury through the promotion of inflammation and cell death [45, 46]. In this context, the inflammatory cytokines and infiltration of immune cells may serve as mediators of the interactions between ferroptosis, apoptosis, and necrosis pathways in steatotic liver subjected to I/R injury.

To investigate the specific mechanisms by which Insig2 regulates ferroptosis in steatosis hepatic I/R, we have identified a molecule—GPX4, which exhibited significant changes at both the transcriptional and protein levels from the sequencing data. Ferroptosis is regulated by cyst(e)ine/GSH/GPX4 antioxidant pathway, and GPX4 plays a cornerstone role in protecting cells by reducing lipid hydroperoxides to their corresponding alcohols, thereby preventing the propagation of oxidative damage [13, 38]. GPX4-mediated ferroptosis is closely related to the progression of free fatty acid-induced steatosis [47]. Additionally, programmed cell death orchestrated by GPX4-dependent pathways is essential for the pathophysiology of I/R injury in the liver and other organs [48, 49]. Our research first introduced Insig2 as a novel regulator of GPX4 in steatotic hepatic I/R injury. Insig2 could block SREBP processing to maintain lipid homeostasis, which could indirectly affect the level of PUFAs and other oxidative lipids. In vivo oxidative stress levels and SREBP factors could affect the expression of GPX4, thereby regulating the sensitivity of cells to ferroptosis [50, 51].

However, our research also had limitations that were important to highlight. Firstly, we did not elucidate the detailed molecular mechanism by which Insig2 influenced GPX4 expression. We proposed that Insig2/SREBP axis might be responsible to lipid contents remodeling. Secondly, although our preliminary research in steatotic graft was encouraging, further clinical investigations were essential to critically scrutinize the applicability of our findings.

In conclusion, our research found that Insig2 in hepatocytes protected against I/R injury in steatotic liver by regulating GPX4-dependent ferroptosis. Therefore, targeting Insig2/GPX4 axis may be a promising approach of steatotic graft injury treatment.

## Materials and methods

### Animals and study approval

Inbred wild-type (WT) male C57BL/6 mice (4 weeks old) were purchased from Hangzhou Medical College. The Insig2 knock-out (KO) mice of C57BL/6 were kindly provided by Prof. Di Wang, Zhejiang University School of Medicine. All animals were accommodated at 12-h light/dark cycle, with unrestricted access to both water and food. Steatotic liver in mice was induced by feeding with high-fat diet (HFD, 60 kcal% fat) for 8 weeks (Research Diet, D12492, USA). The animal protocols were observed in the guidelines of the National Institute of Health (NIH Publications NO. 88−23, revised in 1996). The experiments were approved by the Zhejiang Provincial People’s Hospital (Affiliated People’s Hospital), Hangzhou Medical College (Approval No. 20250110966929).

### Hepatic I/R surgery

The surgical technique was employed to replicate the graft damage observed in LT. Each experimental group included 4–8 mice. If a mouse died during the surgical procedure, its data were excluded from the analysis. Initially, the hepatic artery and portal vein branches supplying the right and triangular lobes were temporarily occluded using a precision microvascular clamp for a duration of 1.5 h. Subsequently, the clamp was removed to restore blood flow. The sham group performed the same surgery except for the hepatic ischemia. At 6 h post-reperfusion, the animals were euthanized, and both their circulating blood and liver samples were collected for further analysis.

### Liver biochemical measurement and triglyceride determination

Serum alanine aminotransferase (ALT) and aspartate aminotransferase (AST) were measured by Chemray 800 chemistry analyzer (Shenzhen, China). Serum inflammatory cytokines (*IL-6, IL-1β, and TNF-α*) were quantified by enzyme-linked immunosorbent assay (ELISA) kits (Invitrogen, USA) according to manufacturer’s instructions. The triglycerides (TG) in serum and liver tissues were determined using a Triglyceride assay kit (NJJCBIO, Nanjing, China) according to the protocol.

### Quantitative real‐time PCR and Western blot

Total RNA was isolated from hepatic tissues and cells utilizing the Total RNA Isolation Kit (Vazyme, Nanjing, China), adhering to the protocol provided. One microgram of RNA was converted to complementary DNA (cDNA) using the First Strand cDNA Synthesis Kit (Vazyme, Nanjing, China). Subsequent quantitative real-time PCR (qPCR) analysis was conducted on the ABI QuantStudio 6 Flex platform (Thermo Fisher, USA), employing the SYBR Green PCR Master Mix (Vazyme, Nanjing, China). The mRNA levels were standardized against β-actin expression. Primer sequences for the target genes are detailed in Supplementary Table S1.

Protein extraction from liver samples was carried out using radioimmunoprecipitation assay lysis buffer (Fudebio, Hangzhou, China), with quantification performed via the BCA Protein Assay Kit (Fudebio, Hangzhou, China). The proteins were resolved by SDS-PAGE and then transferred onto polyvinylidene fluoride membranes (Millipore, USA). Membranes were exposed to primary antibodies at 4 °C for an overnight period, followed by incubation with secondary antibodies (Fudebio, Hangzhou, China) for 2 h at ambient temperature. Protein band intensity was assessed using an ECL reagent (Fudebio Hangzhou, China) and Fluorescence Chemiluminescence Image Analyzer (ProteinSimple, USA), with β-actin as a reference protein. A comprehensive list of antibodies utilized is presented in Supplementary Table S2. The original Western blot bands are provided in Supplementary Materials.

### Histological analysis

The steatotic liver tissues from mice fed with HFD were fixed in OCT compound. The degree of steatosis was determined by Oil-Red-O staining. Ischemic liver lobes were processed with 4% paraformaldehyde for fixation, followed by dehydration and paraffin embedding. Sections (4 μm) from the paraffin blocks were subjected to staining with hematoxylin and eosin (H&E), immunofluorescence (IF), and terminal deoxynucleotidyl transferase-mediated dUTP nick-end labeling (TUNEL). Additional methodological details are outlined in Supplementary Information. Histopathological imaging was conducted using a light microscope (OLYMPUS, Japan). A list of antibodies utilized for staining is provided in Supplementary Table S2.

### In vitro hypoxia/reoxygenation (H/R) model

The primary hepatocytes were separated as previously described method—two-step collagenase perfusion [52]. The cells were cultured at serum-free DMEM medium (37 °C, 5% CO_2_) overnight. The steatosis hepatocytes were incubated with oleic acid/palmitic acid (OA/PA, 500 μM/250 μM, Kunchuang Biotechnology, Xian, China) for 2 days. For H/R model, the cells were subjected to hypoxia (94% N_2_, 1% O_2_, and 5% CO_2_) in sugar-free, serum-free DMEM medium for 6 h. Then the cells transferred to normal air conditions in normal DMEM medium for 6 h.

### Small interference RNA (siRNA) transfection

BNL CL.2 cells (Pricella, Wuhan, China) by STR profiling were seeded in six-well plates at 2 × 10^6^ cells/well. After reaching ~70% confluence, the cells were transfected with the indicated siRNA (Tsingke, Beijing, China) using transfection reagent (Yeason, Shanghai, China) for 48 h. The sequences of siInsig2 duplexes were F: 5′- CGGUGUUCGUGGGUAUAAA-3′ and R: 5′- UUUAUACCCACGAACACCG-3′, siInsig1 duplexes were F: 5′- CACUCAGUUUCUUGUGUAU-3′ and R: 5′- AUACACAAGAAACUGAGUG-3′.

### Adeno‐associated‐virus 8 (AAV8) vectors construction

AAV8 vectors for Insig2 knockdown and overexpression were constructed by the Vigene Biosciences Co., Ltd (Shandong, China). The control group received an AAV8-thyroxine-binding globulin (TBG) vector that was devoid of any genetic material. The mice were injected through the tail vein with a viral solution carrying 3 × 10^11^ copies of the AAV8 vector genomes, all in a 100 μL volume.

### Transcriptome, proteome, and metabolome sequencing and analysis

Liver samples from Insig2 KO and controlled WT mice on an HFD feeding that underwent I/R injury were collected for multi-omics analysis. Metware Biotechnology Co., Ltd (Wuhan, China) executed multi-omics sequencing, including sample preparation, extraction, and detection. Transcriptomic and proteomic analyses identified differentially expressed genes/proteins (DEGs/DEPs) with *p* value < 0.05 and fold change > 1.3. Metabolites were differentially expressed based on Variable Importance in Projection (VIP) score > 1 and *p* value < 0.05. Pathway enrichment analysis using the Kyoto Encyclopedia of Genes and Genomes (KEGG) and Gene Ontology (GO) was performed for the DEG/DEPs, with pathways having *p* value < 0.05 deemed statistically significant.

### Detection of iron concentration and oxidative stress indicators

The iron concentration in serum and liver tissue was quantified using the Iron Assay Kit (Solarbio, Beijing, China). The Malondialdehyde (MDA), Glutathione/glutathione disulfide (GSH/GSSG), NADPH/NADP^+^ were measured using Assay Kit (Beyotime, Shanghai, China) according to manufacturer’s instructions.

### Application of ferroptosis inhibitor and agonist

Ferrostatin-1 (Fer-1, Selleck, S7243, USA) and RSL3 (Selleck, S8155, USA) were dissolved in DMSO and subsequently diluted with a vehicle solution composed of PEG300, Tween 80, and normal saline. These compounds were administered intravenously and intraperitoneally at a dosage of 10 mg/kg, 1 h prior to the initiation of I/R surgery, respectively. For the mouse treatment protocol, animals were randomly assigned to either the control or the drug treatment group.

### Statistical analysis

All experiments were performed a minimum of three times and reproducible results were obtained. Data were analyzed and visualized utilizing GraphPad Prism (v9.0), with summary statistics depicted as mean ± standard deviation. For pairwise group comparisons, a two-tailed Student’s *t*-test was applied; while one-way ANOVA with a post hoc test was utilized for assessing differences across multiple groups. Images that typify the average results for each group were carefully selected for presentation. *P* value < 0.05 was considered statistically significant.

## Supplementary information

Supplementary materials

Original western blots

## Data Availability

The data that support the findings of this study are available on request from the corresponding author.
