# Supplementary material for: Insulin-induced gene 2 alleviates ischemia-reperfusion injury in steatotic liver by inhibiting GPX4-dependent ferroptosis
Source: Cell Death Discov. 2025 Apr 1;11:127. doi: 10.1038/s41420-025-02406-y (PMC11962074; doi:10.1038/s41420-025-02406-y)
Supplement: Supplementary file 1 — Supplementary materials [file 41420_2025_2406_MOESM1_ESM.docx]

**Supplementary materials**

**Supplementary Figures**

**
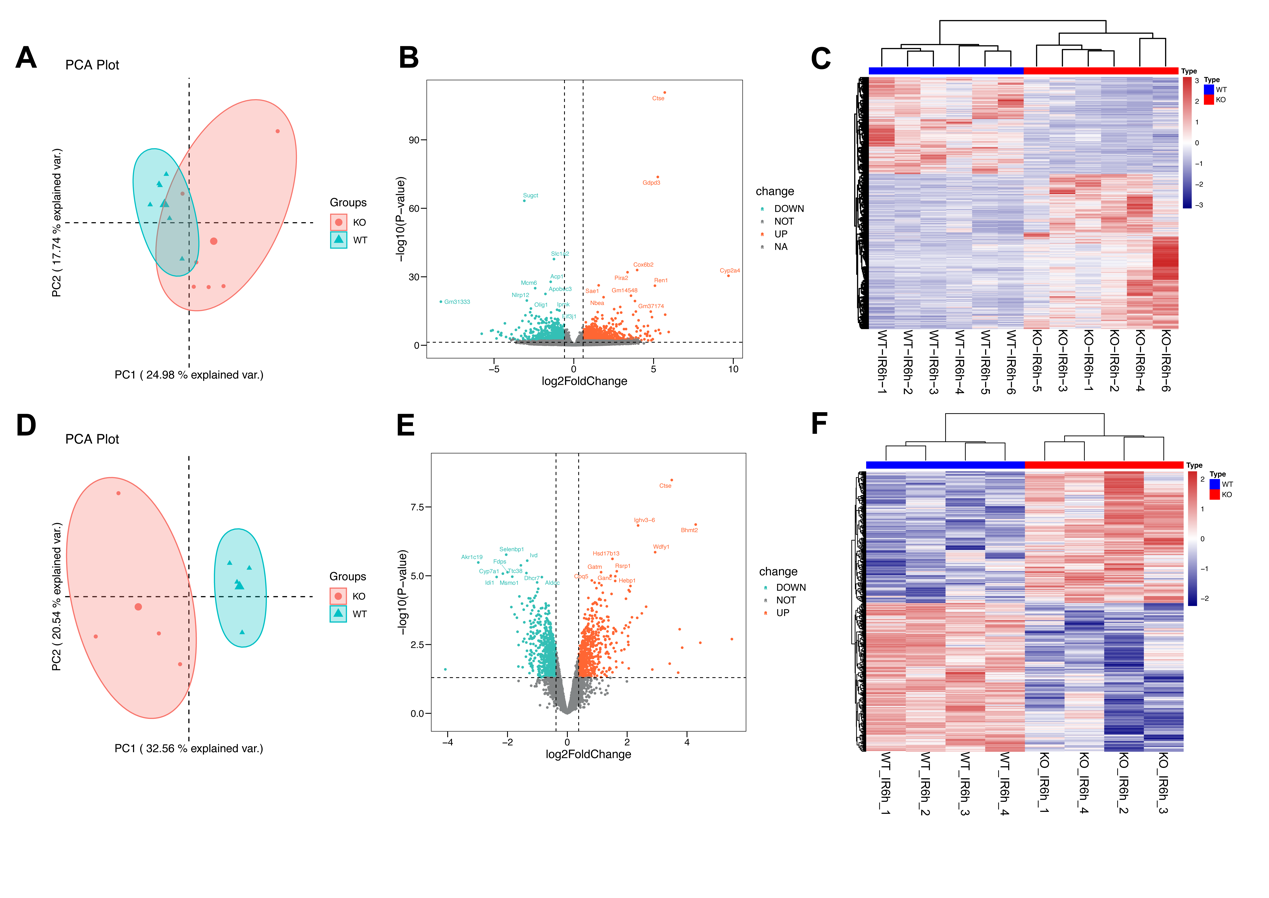
**

**Figure S1.** Transcriptomic and proteomic profiles between WT and Insig2 KO mice with steatotic liver subjected to IR injury. **A-C.** PCA analysis, hierarchical clustering heat map showing global sample distribution profiles, volcano plots indicating DEGs (red, up-regulated genes; blue, down-regulated genes). **D-F.** PCA analysis, hierarchical clustering heat map showing global sample distribution profiles, volcano plots indicating DEPs (red, up-regulated proteins; blue, down-regulated proteins).


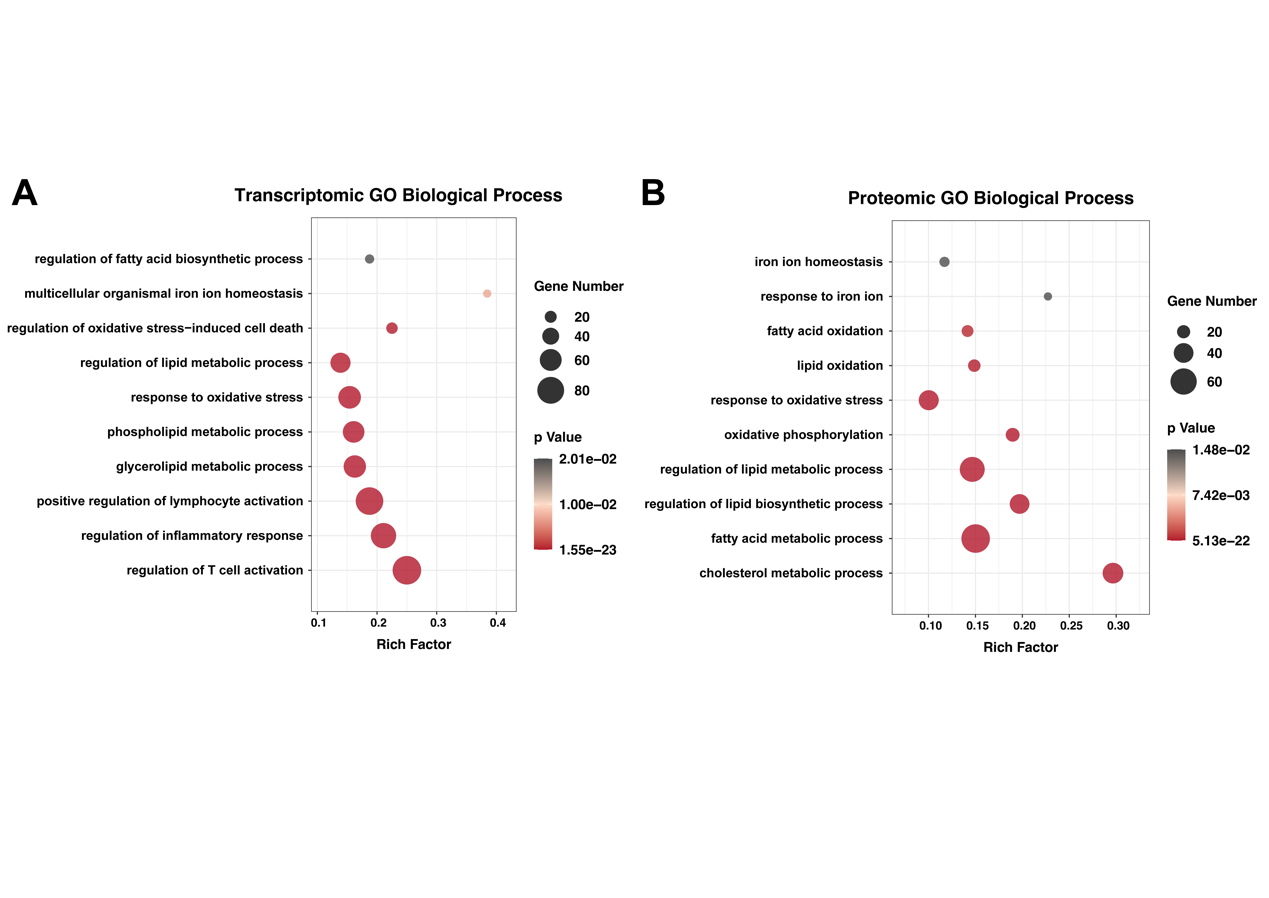


**Figure S2.** GO enrichment analysis reveal key pathways after hepatic IR injury in steatotic liver. **A.** The bubble plot of GO enrichment of transcriptomic results. **B.** The bubble plot of GO enrichment of proteomic results.


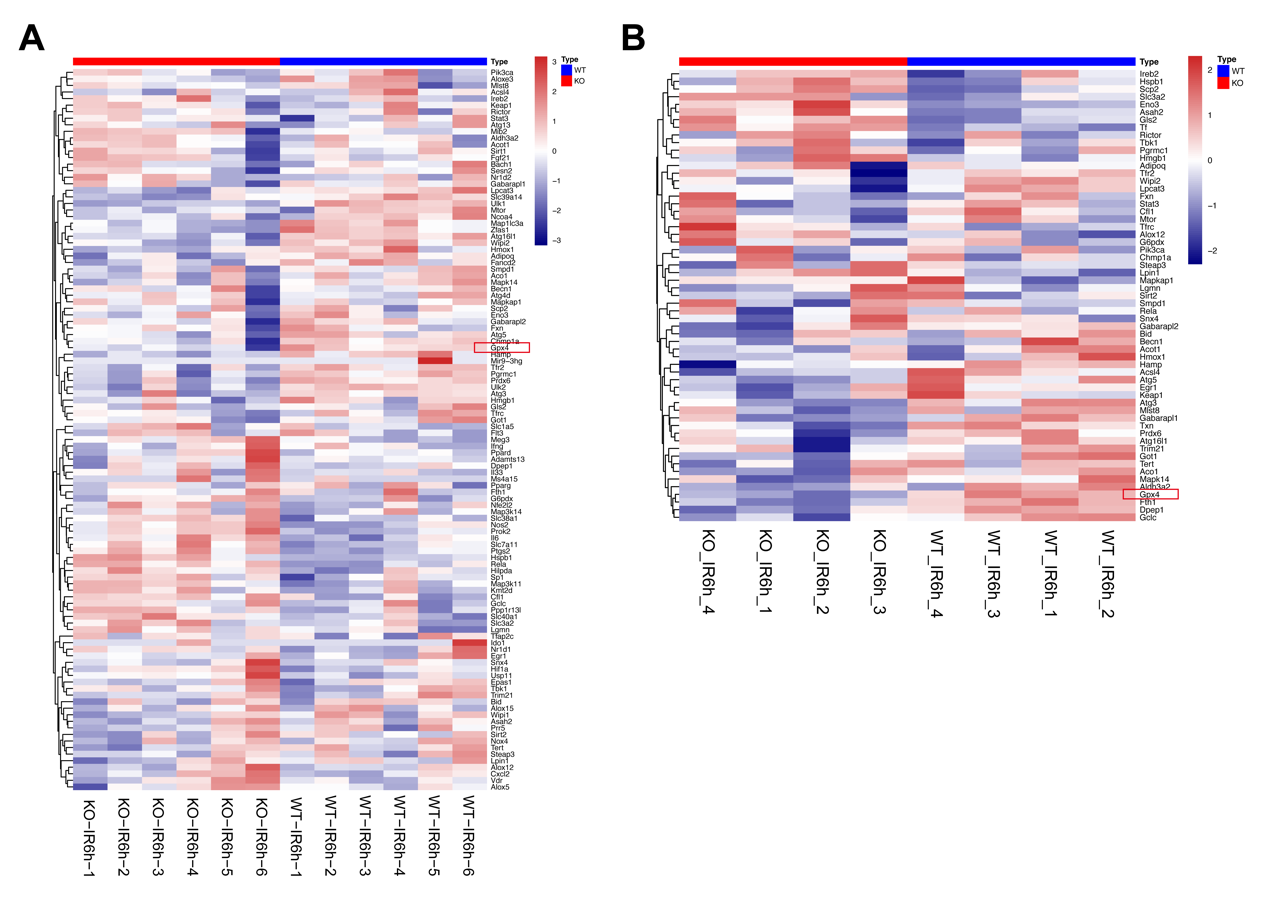


**Figure S3.** The heatmaps of ferroptosis-related genes and proteins between WT and Insig2 KO mice with steatotic liver subjected to IR injury. **A.** The transcriptomic results. **B.** The proteomic results.

**Figure S4.** A diagram showed the experiments of hepatic steatosis, AAV8 transfection, chemical inhibition of GPX4 and hepatic IR injury in C57BL/6 mice.

**
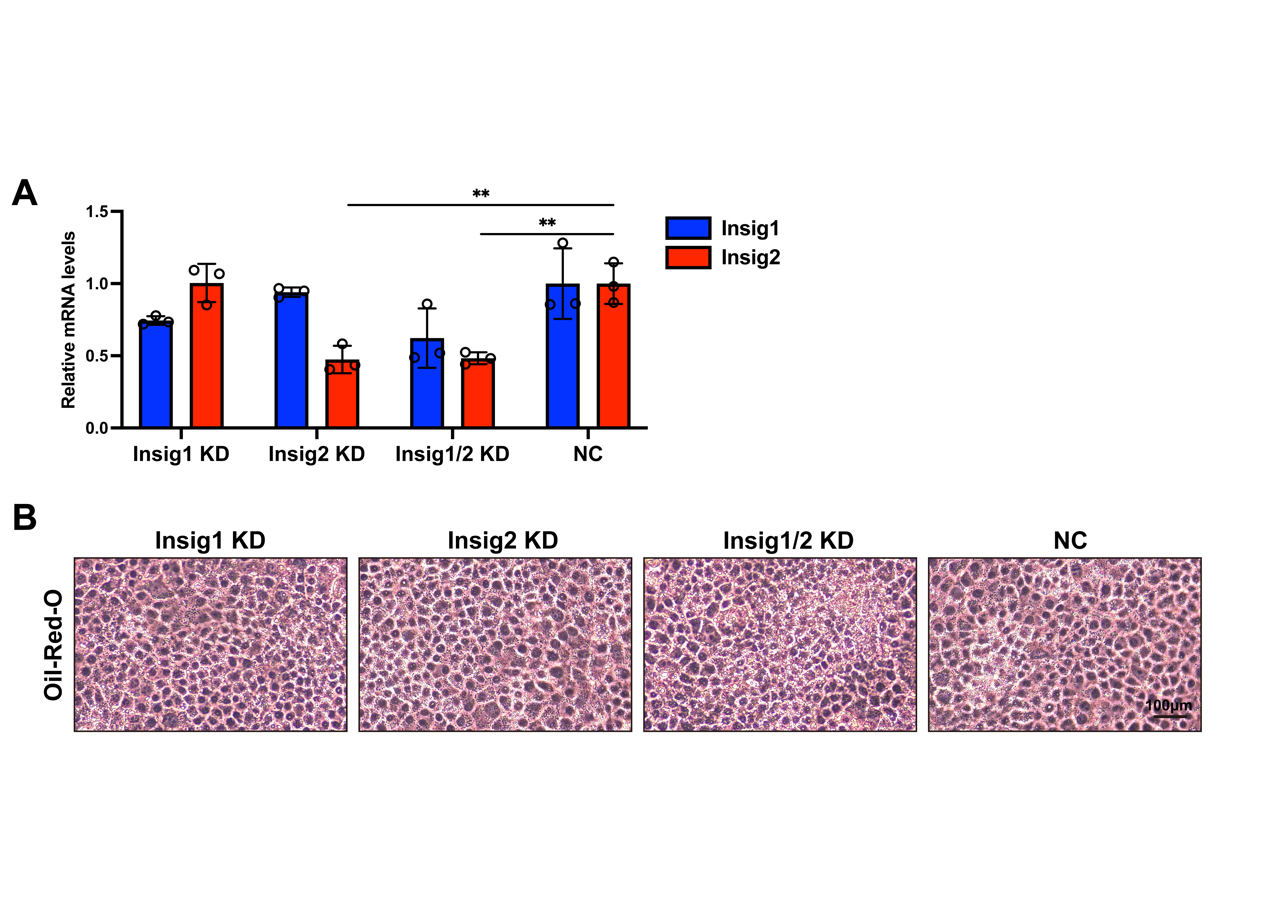
**

**Figure S5.** The complementary role for Insig1 and Insig2 in lipid synthesis. A. siRNA-mediated knockdown of Insig1/2 in BNL CL.2 (mouse liver) cell lines. B. Oil-Red-O staining of cells with single or double knockdown of Insig1/2 after 48-hour incubation with OA/PA (500μM/250μM).

**Supplementary Tables**

**Table S1. Primers for real-time qPCR detection**

| **Gene** |  | **Sequence5'---3'** |
| --- | --- | --- |
| **Mouse Insig2** | **F** | GGAGTCACCTCGGCCTAAAAA |
|  | **R** | CAAGTTCAACACTAATGCCAGGA |
| **Mouse Insig1** | **F** | CACGACCACGTCTGGAACTAT |
|  | **R** | CCAAATGAGAAGAGCACTAGGCT |
| **Mouse IL-6** | **F** | CTGCAAGAGACTTCCATCCAG |
|  | **R** | AGTGGTATAGACAGGTCTGTTGG |
| **Mouse IL-1β** | **F** | TTCAGGCAGGCAGTATCACTC |
|  | **R** | GAAGGTCCACGGGAAAGACAC |
| **Mouse TNF-α** | **F** | CAGGCGGTGCCTATGTCTC |
|  | **R** | CGATCACCCCGAAGTTCAGTAG |
| **Mouse Ccl2** | **F** | TAAAAACCTGGATCGGAACCAAA |
|  | **R** | GCATTAGCTTCAGATTTACGGGT |
| **Mouse Cxcl10** | **F** | CCAAGTGCTGCCGTCATTTTC |
|  | **R** | GGCTCGCAGGGATGATTTCAA |
| **Mouse Bax** | **F** | AGACAGGGGCCTTTTTGCTAC |
|  | **R** | AATTCGCCGGAGACACTCG |
| **Mouse Bcl2** | **F** | GCTACCGTCGTGACTTCGC |
|  | **R** | CCCCACCGAACTCAAAGAAGG |
| **Mouse GPX4** | **F** | TGTGCATCCCGCGATGATT |
|  | **R** | CCCTGTACTTATCCAGGCAGA |
| **Mouse PTGS2** | **F** | TTCCAATCCATGTCAAAACCGT |
|  | **R** | AGTCCGGGTACAGTCACACTT |
| **Mouse Alox12** | **F** | ACCTCAGACAATAGCAGCGGA |
|  | **R** | TCAACGTCCATTCAAAGTCCAG |
| **Mouse** **β-actin** | **F** | GTGACGTTGACATCCGTAAAGA |
|  | **R** | GCCGGACTCATCGTACTCC |

**Table S2. Antibodies for immunoblot analyses**

| **Antibody** | **Cat No.** | **Manufacture** |
| --- | --- | --- |
| **Insig2** | 24766-1-AP | Proteintech |
| **Bax** | 2772S | CST |
| **Bcl2** | ab182858 | Abcam |
| **Cleaved-casepase 3** | 19677-1-AP | Proteintech |
| **F4/80** | 28463-1-AP | Proteintech |
| **MPO** | 22225-1-AP | Proteintech |
| **β-actin** | 66009-1-Ig | Proteintech |

**Supplementary information**

**Hematoxylin and eosin (H****&E) staining**

The H&E staining process involved deparaffinization, rehydration, staining with hematoxylin to color the nuclei, followed by eosin to stain the cytoplasm and extracellular matrix, then dehydrating, clearing, and finally mounting the sections with a non-aqueous medium to examine cellular structures under a microscope.

**Immunofluorescence (IF) staining**

Formalin-fixed and paraffin embedded tissues were also sectioned and stained for F4/80 and MPO IF staining. The process involved antigen retrieval, peroxidase blocking, primary antibody incubation, secondary antibody incubation, mounting the sections. In this study, primary antibodies targeting mouse MPO (22225-1-AP, Proteintech, Wuhan, China) and F4/80 (28463-1-AP, Proteintech, Wuhan, China) were employed. Furthermore, a secondary antibody, specifically goat anti-rabbit/mouse IgG-HRP (HKI0005, Haoke, Hangzhou, China), was utilized.

**Terminal deoxynucleotidyl transferase-mediated dUTP nick-end labeling (TUNEL) staining**

According to the manufacturer’s instructions, paraffin-embedded sections were subjected to deparaffinization, antigen retrieval. After rinsing twice with PBS, TUNEL working solution (HKI0008, Haoke, Hangzhou, China) was added to the reaction mixture and incubated at 37°C for 1 hour. Finally, sections were rinsed three times with PBS before DAPI staining.
