# Supplementary material for: Insulin-induced gene 2 alleviates ischemia-reperfusion injury in steatotic liver by inhibiting GPX4-dependent ferroptosis
Source: Cell Death Discov. 2025 Apr 1;11:127. doi: 10.1038/s41420-025-02406-y (PMC11962074; doi:10.1038/s41420-025-02406-y)

Figure 1D.

Insig2:

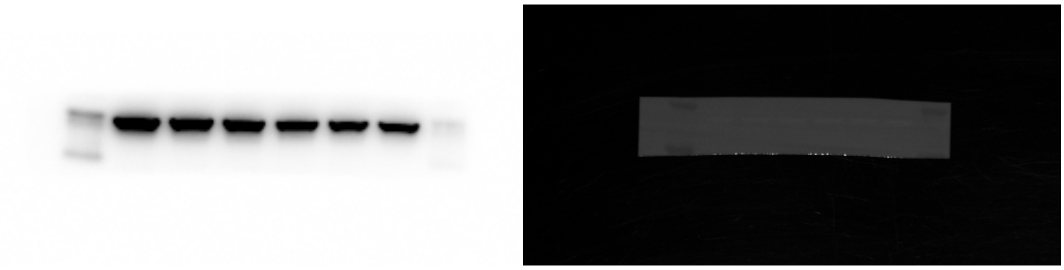

Actin:

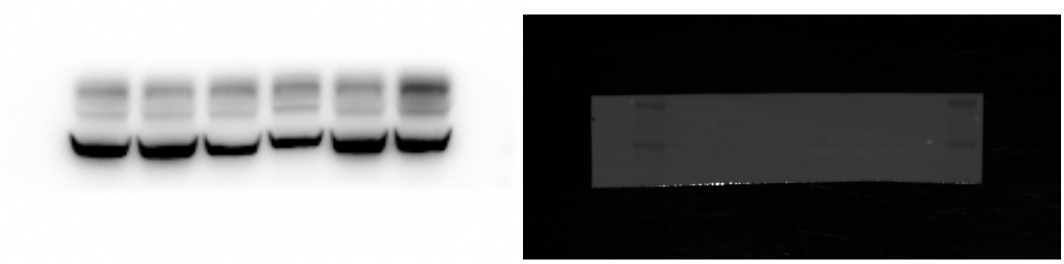

Figure 1E.

Insig2:

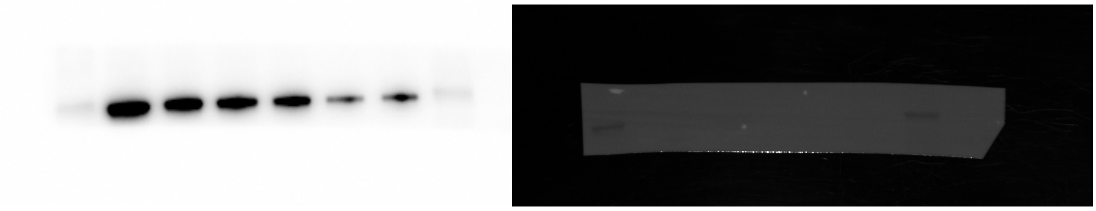

Actin:

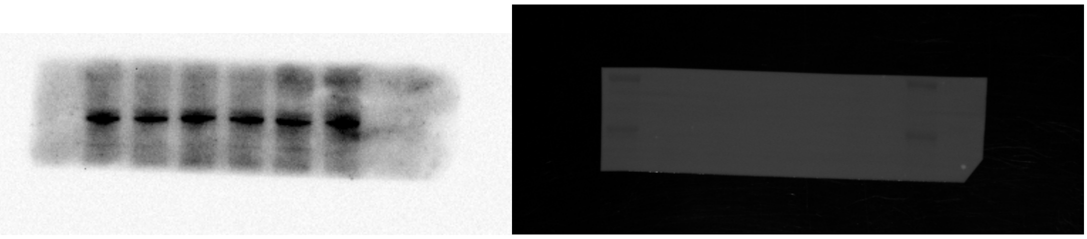

Figure 2G.

Bax

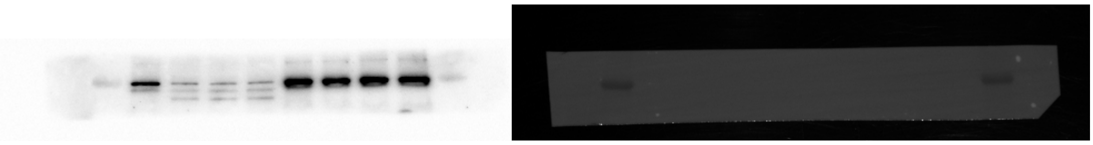

Bcl2

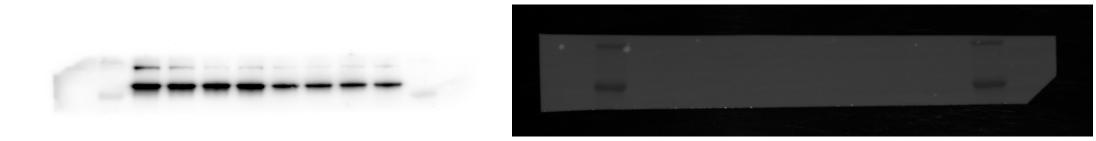

Actin

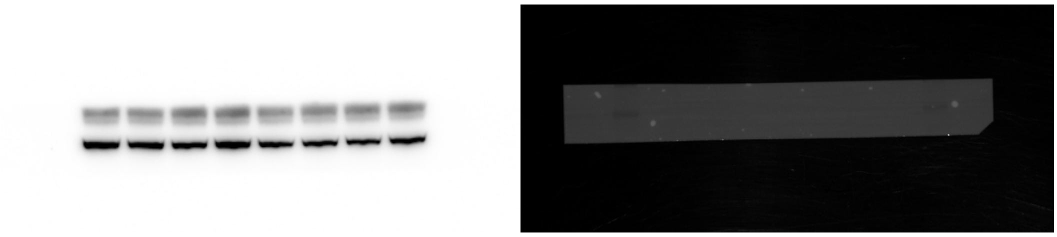

Figure 3A.
Insig2

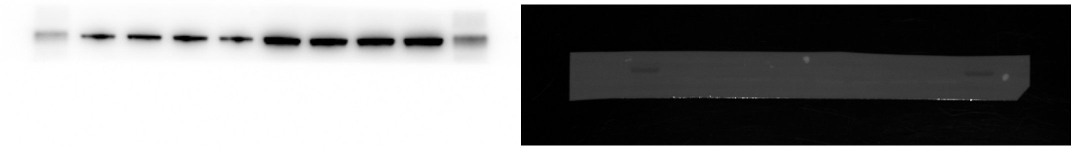

Actin

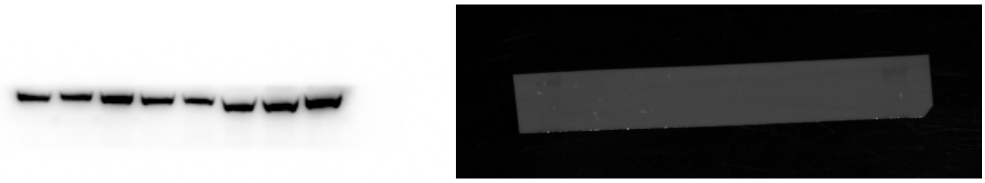

Figure 3H.
Bax

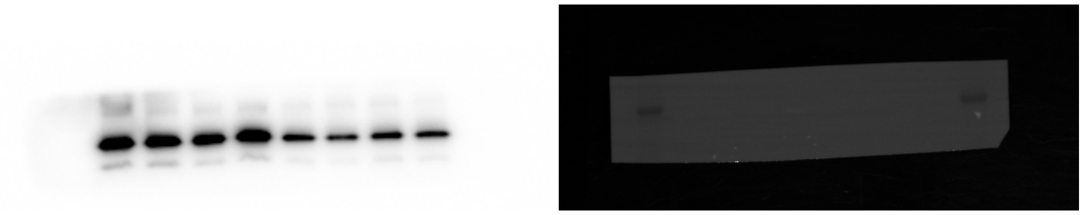

Bcl2

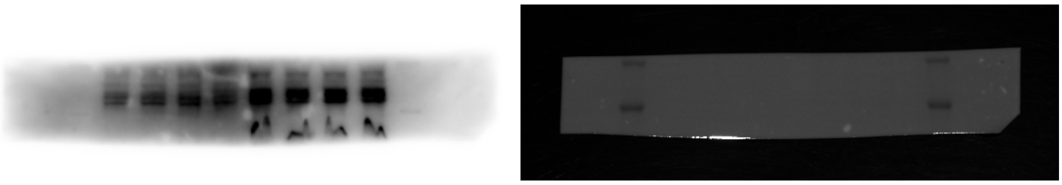

Actin

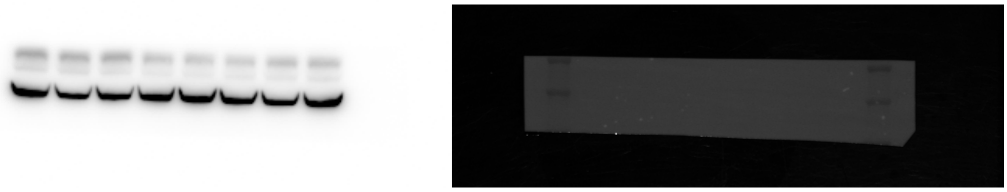

Figure 4A.
Insig2

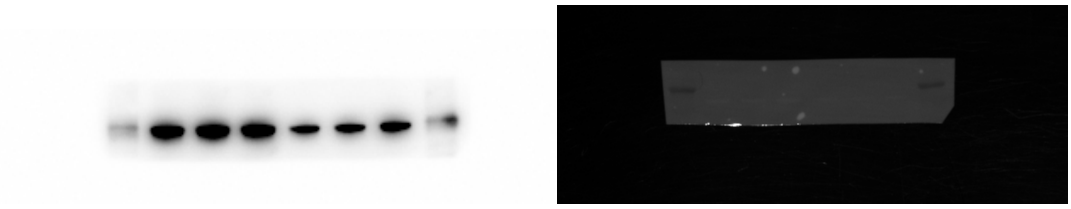

Actin

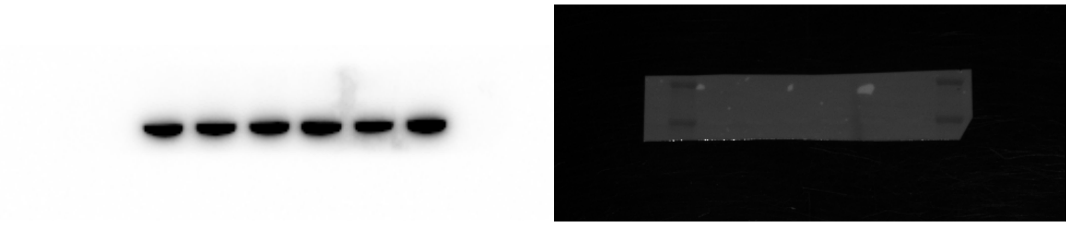

Figure 4C.
Bax

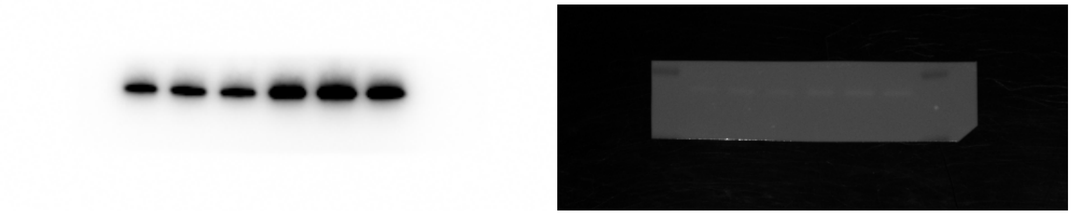

Bcl2

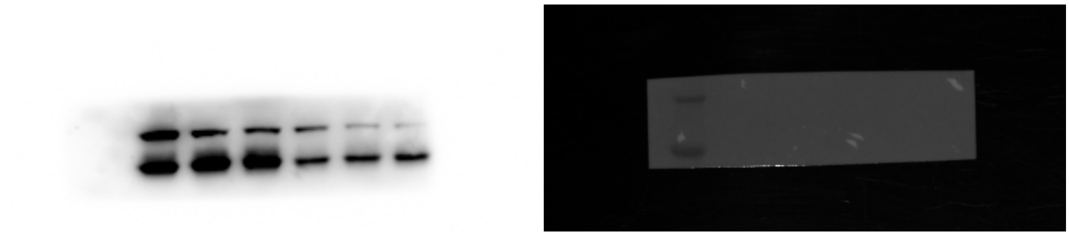

C-casepase 3

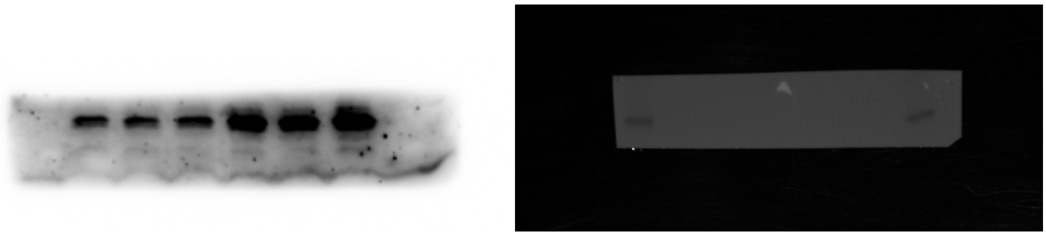

Actin

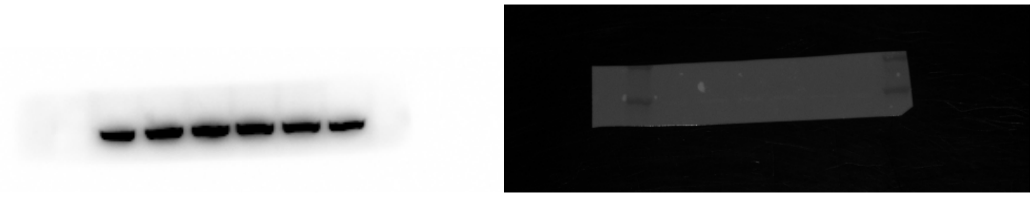

Figure 4D.
Insig2

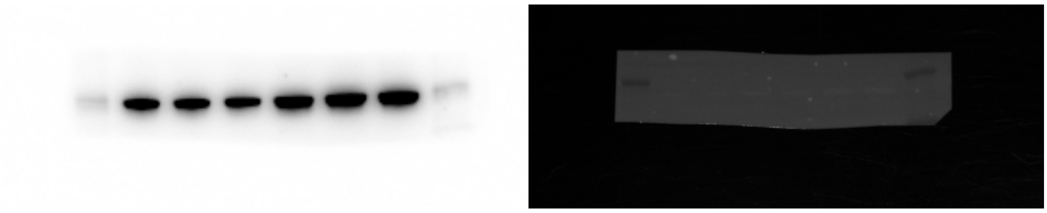

Actin

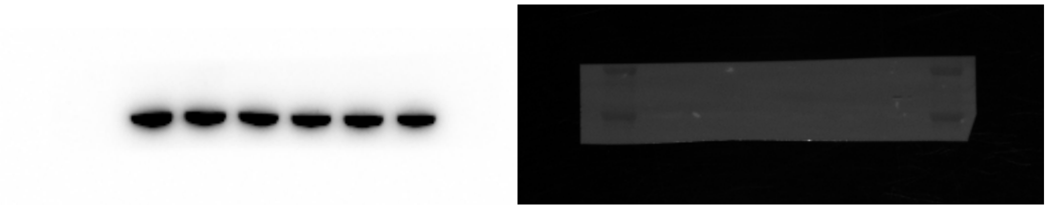

Figure 4F.

Bax

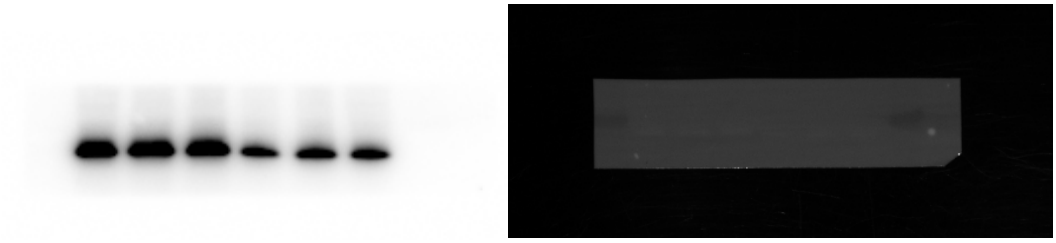

Bcl2

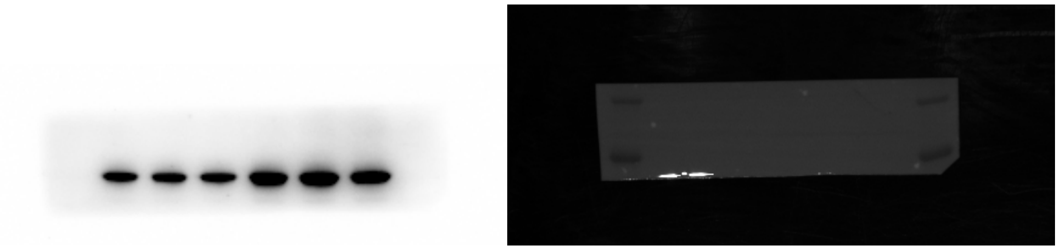

Actin

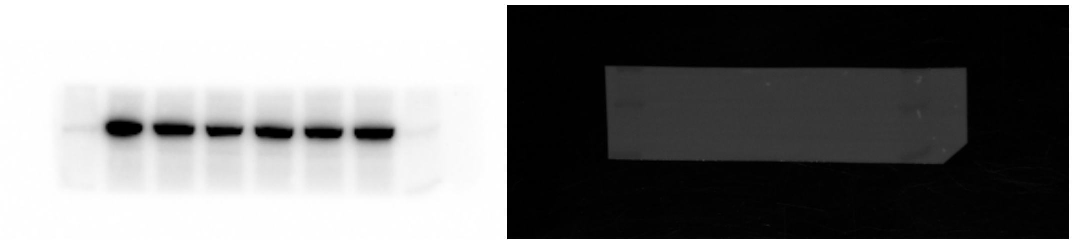

Supplement: Supplementary file 2 — Original western blots [file 41420_2025_2406_MOESM2_ESM.pdf]
